# Mucosal immunity: The missing link in comprehending SARS-CoV-2 infection and transmission

**DOI:** 10.3389/fimmu.2022.957107

**Published:** 2022-08-17

**Authors:** Michael W. Russell, Jiri Mestecky

**Affiliations:** ^1^ Department of Microbiology and Immunology, Jacobs School of Medicine and Biomedical Sciences, University at Buffalo, Buffalo, NY, United States; ^2^ Department of Microbiology, Heersink School of Medicine, University of Alabama at Birmingham, Birmingham, AL, United States; ^3^ Laboratory of Cellular and Molecular Immunology, Institute of Microbiology of the Czech Academy of Sciences, Prague, Czechia

**Keywords:** mucosal immunity, immunoglobulin A, COVID-19, SARS-CoV-2, infection, transmission

## Abstract

SARS-CoV-2 is primarily an airborne infection of the upper respiratory tract, which on reaching the lungs causes the severe acute respiratory disease, COVID-19. Its first contact with the immune system, likely through the nasal passages and Waldeyer’s ring of tonsils and adenoids, induces mucosal immune responses revealed by the production of secretory IgA (SIgA) antibodies in saliva, nasal fluid, tears, and other secretions within 4 days of infection. Evidence is accumulating that these responses might limit the virus to the upper respiratory tract resulting in asymptomatic infection or only mild disease. The injectable systemic vaccines that have been successfully developed to prevent serious disease and its consequences do not induce antibodies in mucosal secretions of naïve subjects, but they may recall SIgA antibody responses in secretions of previously infected subjects, thereby helping to explain enhanced resistance to repeated (breakthrough) infection. While many intranasally administered COVID vaccines have been found to induce potentially protective immune responses in experimental animals such as mice, few have demonstrated similar success in humans. Intranasal vaccines should have advantage over injectable vaccines in inducing SIgA antibodies in upper respiratory and oral secretions that would not only prevent initial acquisition of the virus, but also suppress community spread *via* aerosols and droplets generated from these secretions.

## The problem

In the first place, SARS-CoV-2 is an infection of the upper respiratory tract (URT) mucosae, i.e., the nasal passages and oropharynx. Only later, when, or indeed if, the virus reaches the lower respiratory tract (LRT) and lungs does it cause the severe acute respiratory syndrome known as COVID-19. It is an airborne infection mostly acquired by inhalation of virus-containing droplets and aerosols into the nose or mouth, or *via* the conjunctiva of the eyes and drainage into the nasal passages through the lacrimal duct. Enteric infection can also occur, although the quantitative impact of this in the current pandemic is uncertain (1), and it remains predominantly a respiratory infection (2). Interestingly, however, patients with gastrointestinal infection have been reported to have a better clinical outcome (3). There is little or no hematogenous spread, at least until advanced COVID develops, when viral RNA may become detectable in the circulation (4). Consistently with its URT location, infection is monitored almost exclusively by nasal swabbing.

Secondly, community transmission occurs by the emission of droplets and aerosols containing the virus mainly from the mouth during normal speech, exacerbated by sneezing and coughing, vigorous breathing associated with exercise, and by shouting or singing (5, 6). The vehicles of such transmission are the saliva and secretory fluids of the URT. However, these are not merely passive carriers, as they contain a wide variety of anti-microbial factors, including antibodies mainly of the secretory IgA (SIgA) type. Although inadequately explored, it is reasonable to expect that such factors will have a significant impact on the infectivity of the emitted particles.

These two sets of facts should elicit serious attention to *mucosal immunity* against SARS-CoV-2 (7), yet immunologists have been focused almost exclusively on the evaluation of circulating antibodies, predominantly of the IgG isotype, on cytotoxic T cells, and to a lesser extent on innate mechanisms of immunity. This has been driven in part by the need to develop, as rapidly as possible, treatments as well as vaccines to forestall serious disease and death. To a large extent, this has now been accomplished by the extraordinarily rapid development of several injectable vaccines, which are having a major effect on the outcomes of the pandemic.

However, prevention of serious disease and death may be insufficient in itself to control the pandemic. For this to be achieved, it is essential to *suppress community transmission of the virus*. While public health interventions of masking, social distancing, increased ventilation, etc., have an important part to play, immunological control of transmission will require the induction of anti-viral antibodies in the respiratory and oral secretions, which are the source of the infective droplets and aerosols (6). A key question in this context is: Why do systemically immunized subjects continue to have the virus in their salivary and nasal secretions (8, 9).

It is the purpose of this article to discuss the reasons for this puzzling finding, on the basis of what is already known about the *mucosal immune system* and how it differs from the circulatory immune system, with particular reference to the response to SARS-CoV-2 infection. We further consider how mucosal immunity might be exploited by appropriate immunization strategies not only to prevent infection and disease but also to suppress transmission of SARS-CoV-2.

## Separate and independent: mucosal and systemic immunity

Studies of immune responses in the circulation and in external secretions, including both antibodies as well as the cells involved, reveal that the systemic and mucosal compartments of the immune system are distinct and largely independent (10). This is particularly evident from original comparative studies of antibodies present in secretions and plasma (**Table 1**). Antibody responses induced in secretions are quite distinct from those in plasma with respect to their respective origins, levels, isotypes, specificities, and functions.

**Table 1:** Comparative properties and differences between mucosal (oral and nasal) and systemic immune compartments.

	Nasal	Oral	Systemic
Concentrations (µg/ml)			
IgG	8 – 304^a^	1 – 42^b^	7,000–13,000
IgM	?^c^	64	500–2,500
IgA	70 – 846	194 – 206	500–3,500
IgA subclasses (%)			
IgA1	93^d^	63	85
IgA2	7	37	15
IgA molecular forms (%)			
Polymer	?^e^	96	1–5
Monomer	?^e^	4	95–99
Sites of IgA production	nasal mucosa	salivary glands	bone marrow (spleen) (lymph nodes)
	**Mucosal**		**Systemic**
Production (mg/kg/day)			
IgA	~50		~22
Metabolism	selective transport		catabolized in liver
Circulatory half-life (days)			
IgA1	NA^f^		6
IgA2	NA^f^		4.5
Maturation (years)	1 – 2 (adult levels in infancy)		14 – 20 (adult levels in adolescence)
Effector functions	inhibition of absorption antigen neutralization inhibition of binding to epithelia		anti-inflammatory antigen neutralization

a Dependent on method of collection.

b Dependent on gingival crevicular fluid.

c Not reported.

d Based on % of IgA1- or IgA2-secreting cells.

e Not established.

f Not applicable.

Systemic immunization of individuals with previously unencountered (novel) antigens induces immune responses that are measurable in serum and peripheral blood cells, but usually not in secretions (11–13). In contrast, mucosal immunization induces mucosal responses but not parallel systemic immune responses, thereby demonstrating a considerable degree of mutual independence (11–13). However, antigens previously introduced by a mucosal route can also prime the immune system so that subsequent systemic immunization induces both systemic and mucosal antibody responses (14, 15). There are several examples of the effectiveness of systemic immunization of individuals who were previously exposed to a particular antigen at a mucosal surface for the generation of mucosal responses. Systemic immunization of adults with influenza virus or pneumococcal polysaccharides, which are commonly encountered in early childhood, induces IgA and IgG immune responses in nasal washes or saliva (14, 16). Although systemic immunization with COVID vaccines in uninfected individuals does not generate mucosal antibodies in secretions, infection with SARS-CoV-2 induces antibodies in several secretions (17–23).

Initial exposure to a previously unencountered antigen by a mucosal route (oral or nasal) induces specific SIgA antibody responses both locally and at remote mucosal sites, as well as T cell-mediated systemic unresponsiveness termed “mucosal tolerance” (24). Such diminished systemic responsiveness of T cells can interfere with the generation of protective T cell-dependent immunity. Mucosal tolerance has been amply demonstrated in numerous animal models (25) and also in humans (26), and it is more readily induced by soluble than by particulate antigens. Importantly, individuals with pre-existing, systemically acquired immune responses are refractory to the induction of mucosal tolerance (27). While mucosal tolerance might result in diminished systemic T cell responses after mucosal immunization of naïve individuals, this is unlikely to occur after prior systemic immunization or infection. Hence the temporal sequence of immunization and exposure to infection is critically important in vaccination efforts.

External secretions of the nasal mucosa and the oral cavity (saliva) contain IgA predominantly (90-95% or more) in its secretory form, composed of 2 or 4 monomeric (m) IgA units, J chain, and secretory component (28). Although the collection methods significantly affect the levels of total IgA measured in saliva and nasal secretions, IgA concentrations are nevertheless highly variable within and between individuals and are much lower than those found in plasma (**Table 1**). This is also true for IgG and IgM. The variability of total Ig concentrations must be taken into account in the quantitative evaluation of specific antibodies in secretions, and antibody levels should be expressed relative to the total level of the Ig isotype.

The difference between mucosal and systemic compartments extends to the cellular source of Igs in secretions and plasma. Plasma IgA is present almost exclusively in monomeric form, with a pronounced dominance of the IgA1 subclass, and is produced mainly in the bone marrow (29, 30), whereas mucosal IgA is produced in polymeric form (mainly dimers and tetramers) by numerous plasma cells underlying the epithelium. The epithelial cells express polymeric Ig receptor (pIgR), which transports locally synthesized, J chain-containing, polymeric (p) IgA across the epithelium into the external secretion, and is proteolytically cleaved during transcytosis to release its extracellular domain bound to pIgA as the secretory component of SIgA (31). Contrary to the common but erroneous belief that circulating plasma Igs of any isotype contribute significantly to specific antibodies in external secretions, several studies have clearly demonstrated that this is not true (32, 33). Systemic immunization with poliovirus or rubella virus induces corresponding antibodies in the circulation but not in secretions, whereas oral or intra-nasal immunization achieves the converse: antibodies, mostly of the IgA isotype, induced in intestinal, salivary, and nasopharyngeal secretions (11). The systemic Pfizer mRNA vaccine has been reported to induce weak IgA responses in saliva and nasal secretions especially in previously infected subjects (20, 34–36), interestingly with a predominance of the IgA1 subclass (37) which is susceptible to cleavage by bacterial IgA1 proteases (38). Unfortunately, there are no available data to compare these findings with mucosally delivered SARS-CoV-2 vaccines to determine if results similar to those obtained with polio or rubella vaccines are valid also for SARS-CoV-2, although there is no reason to think otherwise.

Other studies also clearly show that circulating IgG, IgM, and pIgA or mIgA are not effectively transported into secretions. Radiolabeled Igs injected intravenously appeared only in trace quantities (~1% of total corresponding isotype) in external secretions (32). Quantitative assay of Igs in the saliva of patients with IgA or IgG multiple myeloma or Waldenström’s IgM macroglobulinemia revealed that only minute quantities of these monoclonal Igs, traced by their idiotypic determinants, reached the saliva (~1% of each isotype present) despite their presence at very high concentration in plasma, and despite the ability of pIgA to bind to pIgR *in vitro* (33). Thus it is clear that plasma Igs are not effectively transported into external secretions. Furthermore, taking into account that levels of Igs in saliva and nasal secretion are substantially lower than in plasma (**Table 1**), and that only ~1% of such antibodies are of plasma origin, it must be concluded that circulating specific antibodies, irrespective of isotype, cannot provide adequate protection of mucosal surfaces. Importantly these findings provide a rational explanation for the failure of COVID vaccines to suppress viral carriage in systemically immunized recipients (8, 9). In contrast, locally generated mucosal IgA antibodies could be very effective in neutralizing the virus in salivary and nasal secretions, and thereby suppress its transmission to other individuals. Unfortunately, few longitudinal quantitative evaluations of antibodies of all major isotypes in plasma, nasal, and oral secretions in relation to the course of SARS-CoV-2 infection have been undertaken. However, nasal IgM, IgG, and IgA antibodies to spike and nucleocapsid proteins have been found to increase within a few days after infection, and were associated with lower viral loads and with resolution of symptoms (39). Mucosal IgA antibody responses were developed against spike protein within 4 days of SARS-CoV-2 infection in conjunctival and nasal fluids of children and adults (40). Children who remained asymptomatic and cleared the virus earlier had higher levels of IgA antibodies in nasal fluids and later developed higher levels of plasma IgG antibodies than those who became symptomatic. In adults, an early development of serum IgA antibodies was associated with mild disease. These findings have been recently supported by similar results reported in a pre-print (41), and suggest an important role for mucosal IgA antibodies in the URT in determining the course of infection.

Circulating antibodies that do not reach the URT secretions in significant quantities can have only a minimal effect against initial acquisition and infection by a respiratory virus such as SARS-CoV-2. It is regrettable that mucosal immune responses have not been accorded the attention necessary for adequate evaluation of immunity to SARS-CoV-2 infection (7). The majority of published studies focusing solely on plasma antibodies have only limited value in the evaluation of protective immunity against *infection* as opposed to *disease*.

## Circulating antibodies are irrelevant to mucosal protection

It should by now be abundantly clear that measuring antibodies in serum does not reflect responses in mucosal secretions. Yet surprisingly, evaluations of antibody responses to COVID-19 have focused almost exclusively on serum, despite the fact that SARS-CoV-2 initially infects the mucosae of the URT. Nevertheless, whenever looked for, mucosal IgA antibodies to SARS-CoV-2 antigens have been repeatedly detected in secretions including saliva, nasal fluids, tears, tracheo-bronchial secretions, and even breast milk of subjects infected with the virus (17–23). However, missing from most of these evaluations are satisfactory procedures for the *quantitative* assay of antibodies in secretions, when the baseline concentrations of Igs are so variable as a result of inherent factors as well as arising from the method of collection (see **Table 1**) (42).

IgG antibodies to SARS-CoV-2 antigens have been frequently reported in secretions such as saliva, but their quantification is compromised by inappropriate means of collection, including the use of oral swabs that increase the contribution of gingival crevicular fluid containing plasma-derived proteins (42). In addition, assay of antibodies by end-point titer or other uncalibrated procedure ignores the baseline concentration of the Ig being assayed, unless total Ig isotype is also measured. IgG is only a very minor component of the Igs present in glandular saliva (~1µg/m) compared to up to ~200µg/ml of IgA, which is mostly in the form of SIgA. Almost all the IgG found in whole saliva is derived from the circulation *via* gingival crevicular fluid (43, 44), the amount of which increases with gingival inflammation (45). Most adults >35 years of age have some degree of periodontal inflammation (46), which results in increased gingival crevicular fluid flow, and even the acts of chewing, as well as massaging the gums, tooth-brushing, and oral swabbing, enhance the transudation of tissue fluid containing plasma-derived Igs from the gingival sulcus into saliva. In addition, the concentration of IgA in saliva is inversely related to flow-rate (47), thus enhancement of salivary flow, either inadvertently or through the use of stimulants to facilitate collection, distorts the assay of specific antibodies unless correction for this is applied. Unfortunately, these factors are seldom taken into account in the collection of saliva for analysis of antibodies against SARS-CoV-2, and consequently the results obtained are quantitatively meaningless. Although much less well studied than saliva, which is the most readily accessible secretion, similar considerations doubtless apply to most other secretions. Assay of antibodies in secretions is complicated by the lower and variable concentration of Igs, dependent in part upon idiosyncratic, temporal, and procedural factors, and by difficulties in collecting and handling viscous, mucinous fluids. Unquestionably it is more difficult and less accurate than assay of serum antibodies.

Less is known about the composition of nasal and tracheo-bronchial fluids, but SIgA is the predominant Ig, with smaller amounts of IgG. However, while some IgG may be derived from the circulation by passive transudation (48), there is evidence for local production of IgG (as well as IgD) by plasma cells resident in nasal mucosa (49). Whether the neonatal IgG receptor, FcRn, is expressed in nasal epithelium, as it is in other mucosal sites including bronchial epithelium (31, 50) is unclear, although if so, it might account for the levels of IgG in nasal as well as tracheo-bronchial secretions. As these are usually collected either by swabbing or lavage, again assays of respiratory antibodies may not be quantitatively reliable unless allowance is made for the method of collection and the dilution of sample obtained. Note that the secretions of the deep lung tissue, i.e., the terminal airways and alveoli, are quite different, lacking SIgA and instead containing IgG (and mIgA) derived from the circulation. Thus a gradient exists along the respiratory tract, from an essentially mucosal characteristic in the URT to an essentially serosal characteristic in the lungs.

## Primacy of mucosal immunity

**Figure 1:**
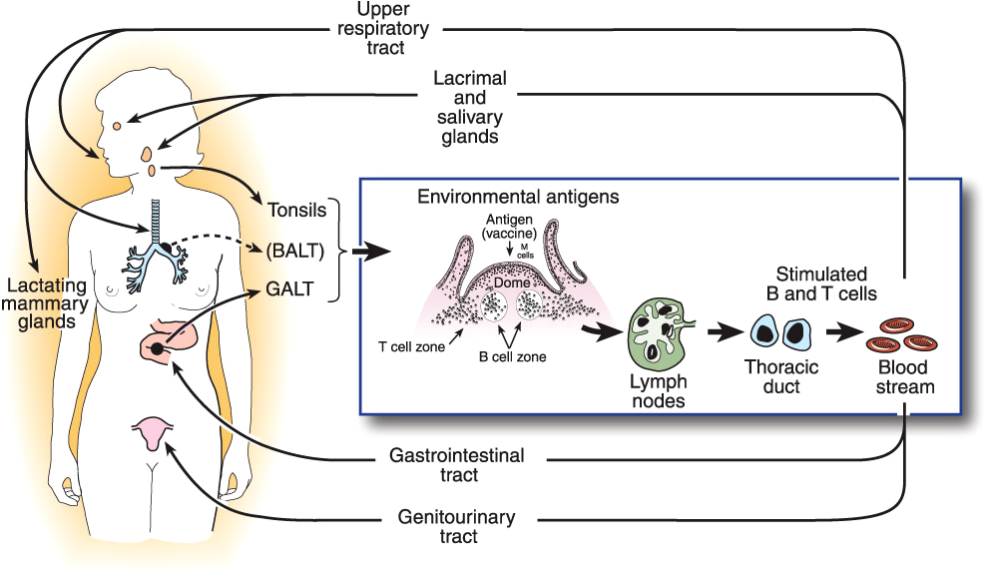
The common mucosal immune system, illustrating the origin of antigen-stimulated, IgA-committed B cells and cognate T cells in inductive sites, mainly the organized mucosa-associated lymphoid tissues of the respiratory and intestinal tracts, i.e., the palatine, tubal, and lingual tonsils and adenoids, and the gut-associated lymphoid tissues (GALT) i.e., small intestinal Peyer’s patches and large intestinal follicles, respectively. Note that bronchus-associated lymphoid tissue (BALT) does not usually occur in healthy adults, but can be found in children, or be induced by infection (52). Antigen-stimulated B and T cells emigrate and traffic through mesenteric lymph nodes into the circulation, and ultimately home into the lamina propria of respiratory, intestinal, and genital tracts and stroma of salivary, lacrimal, and lactating mammary glands, etc. Homing of cells into mucosal effector sites is orchestrated by the expression of vascular endothelial addressins and production of chemokine ligands in mucosal effector sites, and corresponding integrins and chemokine receptors expressed on B and T cells induced in mucosal inductive sites. Terminal differentiation of B cells into pIgA-secreting plasma cells occurs in these effector sites with help from T cells and locally produced cytokines. SIgA is formed by the pIgR-mediated epithelial transport of this locally synthesized pIgA into the secretions, the extracellular part of pIgR becoming the secretory component of SIgA.

Quantitative assay of Igs present in all mucosal secretions, including assessment of the total flow rates of these secretions, shows that production of SIgA is by far the most abundant of all Ig isotypes (28), amounting to an estimated 5-10 grams per day in an adult human. This abundance is paralleled by the distribution of immune cells throughout the body: approximately two-thirds of all lymphoid cells (T, B, innate, and their progeny) are located in mucosal tissues, and accessory cells including dendritic cells and phagocytes follow a similar pattern (51). From these simple facts, it may be inferred that protection of the mucosae is the primary quotidian function of the entire immune system operating “24/7/52”. This should not be surprising, given that the great majority of infectious diseases are acquired through the mucosal surfaces which are exposed to the external environment, and that the oral, gastrointestinal, respiratory, and (female) genital tracts are naturally colonized by an extensive microbiota that must be maintained in mutual coexistence with the host, but inhibited from invading (10). This is accomplished by the operation of the common mucosal immune system as outlined above (13) (**Figure 1**).

## Mucosal immunity to SARS-CoV-2 and how it can act against the virus

As noted above, SARS-CoV-2 is a respiratory infection acquired largely by inhalation through the nose or mouth, or *via* the conjunctiva of the eye followed by drainage into the nasal passages. Thus its first contact with the immune system will be through the mucosae of the URT and mucosal inductive sites represented by Waldeyer’s ring of tubal, palatine, and lingual tonsils, and adenoids, located in the pharynx (53, 54). Notably, this is different from the situation in mice, which instead have nasal lymphoid tissue (NALT) covered by follicle-associated epithelium containing M cells underlying the nasopharyngeal tube (55). Although thought to be functionally equivalent for the induction of mucosal IgA antibody responses, the two structures are anatomically and histologically different and are likely to differ in their accessibility to inhaled antigens (56). However, spike-specific germinal center activity has been identified in tonsil biopsies of subjects who had recovered from SARS-CoV-2 infection (57). Isolated lymphoid follicles are present in human nasal mucosa, and may be inducible by infection (49). Dendritic cells are found in the nasal mucosa (58, 59) and may be induced by influenza virus (60). Uptake of viral antigens through these tissues and processing by the underlying immune cells would explain the development of mucosal IgA antibodies in various secretions as a result of the dissemination of sensitized IgA-committed B cells to remote mucosal effector sites within the common mucosal immune system (61–63). If these responses are sufficiently powerful, this could explain why a significant proportion (maybe up to 50%) of SARS-CoV-2 infections remain mild or even asymptomatic, by confining the virus to the URT and preventing its invasion into the LRT where serious COVID develops (39). Results from recent studies evaluating IgA antibody responses to SARS-CoV-2 antigens in relation to the course of infection support this hypothesis (40, 41). Possibly of even greater importance is whether such antibodies in saliva or nasal secretions would neutralize the virus and thereby inhibit its capacity to infect other individuals by the emission of droplets or aerosols containing viral particles. In this connection, it is important to note that pIgA, including SIgA antibodies have been demonstrated to show substantially greater (up to 14-fold) virus-neutralizing activity than mIgA or IgG antibodies of the same specificity (64, 65). Interestingly, hexamerization of an IgG1 monoclonal antibody against SARS-CoV-2 greatly enhances its ability to neutralize the virus (66).

For reasons discussed above, circulating antibodies, predominantly IgG, induced by the currently available systemic vaccines would not be capable of exerting the same protective effects as SIgA antibodies in secretions of the URT, saliva, or tears. These vaccines were designed from the start to induce circulating antibodies that would prevent serious COVID disease and death, and in this they have been hugely successful. In this context it is important to note that the deep lung tissue, including the terminal airways and alveoli, is a completely different environment from the URT, and is dominated by plasma-derived Igs, especially IgG, as well as alveolar macrophages and neutrophils that are recruited during the inflammatory pathology of COVID. Whether systemic vaccines have a major impact on acquisition of the virus and its ability to initiate infection in the URT has not been adequately investigated, and it remains uncertain as variable effects have been reported. Conversely, the frequent occurrence of “breakthrough” infections in vaccinated subjects, and consequent transmission of the virus to other individuals, implies that systemic vaccination is not sufficiently effective in achieving these desirable objectives (67), although this is complicated by the emergence of antigenically diverse variants of SARS-CoV-2. In addition, the oft-cited issue of “original antigenic sin”, whereby subsequent infections with variants of an original virus tend to recall antibodies predominantly against the original viral antigens, diminishes the effectiveness of the response to the new variants (68, 69). However, systemic vaccination of subjects who were previously infected with SARS-CoV-2 results in greater protective immunity to COVID disease as well as much stronger serum IgG antibody responses (70–75), and, most significantly, less capacity to transmit the virus to other individuals (76, 77). This has been mechanistically attributed to “hybrid immunity”, an enhanced condition resulting from a combination of serum antibody-mediated immunity and T cell immune memory plus putative innate mechanisms (78, 79). Unaccountably omitted from consideration is the possibility that prior SARS-CoV-2 infection had primed mucosal immune responses that were recalled by the subsequent systemic vaccination, as noted above (14). Evidence for this is now forthcoming from a report that systemic vaccination (one dose) of previously infected subjects induced the appearance in peripheral blood of IgA anti-spike antibody-secreting cells with mucosal homing potential approximately one week after immunization, as well as IgA antibodies in nasal fluids (15, reviewed by 80), in conformity with the known operation of the mucosal immune system (81). In previously uninfected subjects, IgA antibody-secreting cells of mucosal phenotype were absent from the circulation and nasal IgA antibodies were not induced, even after a second vaccine dose.

## Are mucosal vaccines the answer? Of mice and humans

Although systemic vaccines have been reported to reduce community spread of the virus (82, 83), the mechanisms underlying this effect are unclear. Given that systemic vaccines do not effectively generate antibodies in salivary and nasal secretions, they would be unlikely to inhibit carriage of virus or interfere with transmission to others. On the other hand, diminution of the overall viral load might result in less virus in the mouth and nose regardless of the presence of antibodies in secretions.

Nevertheless, in light of the foregoing discussion it is reasonable to propose that generation of anti-viral antibodies in nasal secretions, saliva, and tears would yield substantial benefit in terms of both preventing initial infection by SARS-CoV-2 and suppressing onward transmission of the virus, neither of which would be readily accomplished by antibodies confined to the circulation (84). This can only be achieved by mucosal routes of immunization that have been demonstrated to induce mucosal antibodies, mainly SIgA, in various human secretions. Historically, several mucosal routes, including oral (enteric), intranasal, rectal, vaginal, and intra-oral (sublingual) have been used experimentally, but only oral and intranasal routes have been approved for human application (63, 85). Among these it is notable that intranasal vaccination has been successfully developed against influenza virus, thereby affording precedent for this approach against SARS-CoV-2. Numerous groups have indeed taken up the challenge and demonstrated that intranasal immunization with a variety of vaccine constructs can induce antibodies with virus-neutralizing capability in secretions as well as serum, and even protective immunity against challenge, in animals such as mice (86, 87), hamsters (88, 89), and monkeys (90, 91, reviewed in 92, 93). However, few of these efforts have advanced into clinical trials beyond phase I. The WHO website shows that of 156 COVID vaccines in clinical development, 8 are intranasal, a further 3 are administered as aerosol or by inhalation, and 4 are orally delivered (https://www.who.int/publications/m/item/draft-landscape-of-covid-19-candidate-vaccines; accessed May 18, 2022). Many efforts at intranasal vaccine development have been abandoned because of failure to repeat preclinical success in phase I trials, a common finding, often the result of inadequate understanding of differences in the human mucosal immune system from those of small animal models (94). It has already been noted above that nasopharyngeal immune anatomy differs between mice (which have NALT in the naso-pharyngeal duct) and humans (which have Waldeyer’s ring of tonsils and adenoids in the pharynx). It has now become clear that inbred, genetically uniform mice of the same age and raised under controlled conditions of hygiene are substantially different in their immune responsiveness from genetically diverse humans exposed to markedly different environmental conditions. Even the body-size difference (~20g for mice vs. ~70kg for humans) relating to the numbers of immune system cells present in each species, and respective lifetimes (~2 vs ~70 years) may represent seldom considered factors. Other differences in IgA physiology relate to the occurrence of two IgA subclasses, a much higher concentration of circulating mIgA1 in humans (95), and absence of the Fcα receptor (CD89) on myeloid cells in mice. Yet the expectation that success in animal models will translate to success in humans all too often proves to be unwarranted and leads to disappointment. It is unfortunate in this regard that negative experimental results and developmental failures are often not reported or are overlooked in the pursuit of success.

While it is clear that live SARS-CoV-2 infection induces mucosal IgA responses, these along with systemic responses tend to decline after a few months (17–23, 39, 96). However, as noted above, prior infection primes the immune system for the recall of responses upon systemic vaccination, including the generation of mucosal IgA antibodies. If this applies also to non-replicating intranasal vaccines, it suggests that the live coronavirus induces significant, potentially protective responses that are not achieved by subunit, mRNA, or even inactivated viral vaccines given intranasally. In this context it is noteworthy that three intranasal COVID vaccines currently in phase III clinical trials are based on replicating viral constructs, either attenuated coronavirus or viral vectors (https://www.who.int/publications/m/item/draft-landscape-of-covid-19-candidate-vaccines; accessed May 18, 2022).

Several experimental COVID vaccines use adenovirus or other viral vectors expressing SARS-CoV-2 spike protein, its receptor-binding domain, or other viral proteins. While some of these have been successful by systemic injection (e.g., Astra-Zeneca, Janssen, and other vaccines), few have met with success as mucosal vaccines. One problem is that adenoviruses are frequently encountered as agents of respiratory infections, causing common cold-like disease, thereby inducing immune responses to these particular viruses that can interfere with their use as vaccine vectors. Indeed an adenovirus-vectored HIV vaccine proved to be counter-effective in clinical trial and had to be abandoned (97). On the other hand, an adenovirus 5-vectored vaccine expressing COVID spike protein has recently been reported to induce mucosal antibody responses in hamsters upon intranasal or oral administration, and to reduce both disease severity and transmission to unimmunized hamsters (89). Phase I clinical trial of this vaccine construct in humans also demonstrated mucosal IgA antibody responses, but whether this leads to protective immunity remains to be seen.

## Conclusions and future prospects: asking the right questions

The above-discussed findings mean that it becomes important to understand the cellular and molecular details of mucosal immune responses induced in humans by actual SARS-CoV-2 infection (98), how these differ from the responses induced by systemically administered vaccines, and why mucosally administered vaccines fail to induce the desired responses in humans when they appear to work in experimental animals. While a few mucosally delivered vaccines have been developed for human use, most are oral and aimed at enteric infections (63). Nevertheless, the success of nasal influenza vaccines indicates that this approach is feasible. The duration of mucosal immune responses and recall of immune memory within the mucosal immune system are inadequately understood issues. Key questions therefore include how mucosal immune responses can be most effectively generated and maintained at protective levels for prolonged periods, or rapidly recalled in the event of infection, in humans, not just in experimental animals. Identification of effective antigenic platforms, appropriate adjuvants, and delivery systems for mucosal vaccines, which will be quite different from those developed for conventional systemic (injected) vaccines, will be important components of success.

## Author contributions

This article was conceived, written, and approved by both authors.

## Conflict of interest

The authors declare that the research was conducted in the absence of any commercial or financial relationships that could be construed as a potential conflict of interest.

## Publisher’s note

All claims expressed in this article are solely those of the authors and do not necessarily represent those of their affiliated organizations, or those of the publisher, the editors and the reviewers. Any product that may be evaluated in this article, or claim that may be made by its manufacturer, is not guaranteed or endorsed by the publisher.
